# Home Treatment for Catarrhs and Colds

**Published:** 1895-09

**Authors:** 


Home Treatment for Catarrhs and Colds. A Handy Guide for the Prevention, Care and Treatment of Catarrhal Troubles, Colds in the Head, Sore Throat, Hay-fever, Hoarseness, Ear Affections, etc. Adapted for use in the household and for vocalists, clergymen, lawyers, actors, lecturers, etc. By Leonard A. Dessar, M. D., Visiting Laryngologist to St. Mark's Hospital and to Mt. Sinai Hospital Dispensary, etc., etc. Illustrated. Pp., 118. New York : Home Series Publishing Co. 1894.

This little book is admirably and concisely written. All parts of it can be read by the student and practitioner with advantage, and many parts of it may be recommended to the layman, such as the preventive treatment of colds and other conditions, the care of the mouth and teeth, hints to vocalists and public speakers and hints on the care of the ears. The author must, however, remember that when he has taught a layman the symptoms and treatment of a simple condition he has not taught him how to distinguish it from a more severe and, probably, a very grave condition and that only the most skilful can do this. It is certainly our duty as physicians to teach preventive medicine. The diagnosis and treatment of diseased conditions can only be carried out by the accomplished physician.

The use of this book in a household, correct as it is in all its statements, would, probably, often illustrate the proverb "a little knowledge is a dangerous thing," for the layman would get but half the truth from its pages. The author, for example, gives the symptoms and appearance of the throat in acute tonsillitis without telling the reader that the patient might have diphtheria, and expects that the treatment laid down will be carried out, when it is admitted that these conditions cannot always be distinguished clinically until a bacteriological examination is made of the excretion. W. S. R.

